# Timosaponin AIII inhibits gastric cancer growth by the promotion of programmed cell death via the activation of p300/acetyl-p53 and Akt/MEK/ERK signaling

**DOI:** 10.3389/fphar.2026.1821689

**Published:** 2026-07-20

**Authors:** Kaizhen Tang, Wairong Zhao, Jing Zhang, Guangyu Wang, Wenting Shi, Meiyan Yue, Yan Lin, Yanli Fu, Zhongyan Zhou

**Affiliations:** 1 Longhua Hospital, Shanghai University of Traditional Chinese Medicine, Shanghai, China; 2 Department of Surgery and Cancer, Faculty of Medicine, Imperial College London, London, United Kingdom; 3 Internal Chinese Medicine Division, The Chinese Medicine Hospital of Hong Kong, Tseung Kwan O, Hong Kong SAR, China; 4 Department of Oncology, The Fourth Affiliated Hospital of Xinjiang Medical University, Urumqi, China; 5 Department of Oncology, Shenzhen (Fu Tian) Hospital, Guangzhou University of Chinese Medicine, Guangdong, China; 6 State Key Laboratory of Pharmaceutical Biotechnology/Department of Pharmacology and Pharmacy, LKS Faculty of Medicine, The University of Hong Kong, Pokfulam, Hong Kong SAR, China; 7 Cardiac and Vascular Center, The University of Hong Kong-Shenzhen Hospital, Shenzhen, China

**Keywords:** gastric cancer, p53, programmed cell death, timosaponin AIII, zebrafish, p300

## Abstract

Gastric cancer (GC) is a common cancer and causes severe deaths worldwide, while the current treatment cannot meet its medical needs. Timosaponin AIII (Timo AIII) is an active component isolated from *Anemarrhena asphodeloides* Bunge, which is a well-known Chinese Materia Medica and has multiple pharmacological activities, particularly anti-cancer activities. In this study, zebrafish and human GC cell models are utilized for the evaluation of the anti-GC activity of Timo AIII by integrating bioinformatics analysis and classical pharmacological approaches. We found that Timo AIII significantly decreased the GC growth in zebrafish. In human GC cell BGC-823, Timo AIII suppressed the cell viability, proliferation and migration in concentration- and time-dependent manners. Timo AIII blocked cell-cycle progression and promoted cell apoptosis. Moreover, Timo AIII activated programmed cell death (PCD), including apoptosis, ferroptosis, necroptosis and autophagy. The pharmacological inhibition of these processes and PI3K/Akt/MAPKs signaling by their specific inhibitors could partially abolish the anti-GC effect of Timo AIII. For the mechanistic study, the bioinformatics analysis revealed that p53 might be the central downstream effector of Timo AIII, promoting PCD. Timo AIII increased the intracellular protein levels of MEK, acetyl-p53 and p300 and the phosphorylation levels of Akt and MEK in BGC-823 cells, while it decreased the protein levels of p53 and Akt and the phosphorylation levels of ERK. In conclusion, Timo AIII presents anti-GC activity and the underlying mechanism is likely to be the activation of PCD via p300/acetyl-p53 and Akt/MEK/ERK signaling.

## Graphical Abstract

**Figure:**
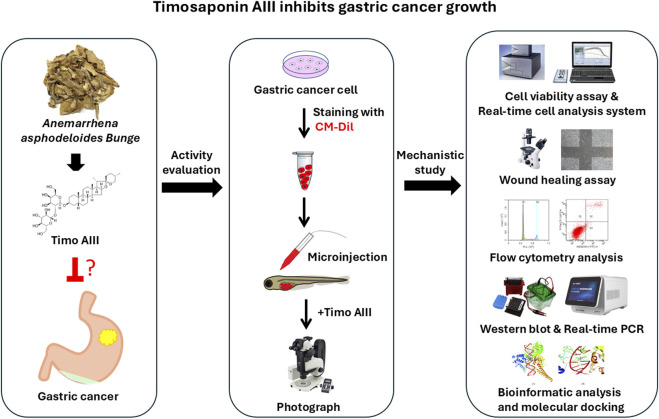

## 1 Introduction

Gastric cancer (GC) is the fifth most common cancer and caused the fifth-highest number of deaths worldwide in 2022 according to Global Cancer Statistics (GLOBOCAN) (Bray et al., 2024). GC is generally rare in adults aged <50 years, and its incidence increases with age (Sung et al., 2021). However, the etiology of GC is not well-understood, and the incidence of GC tends to be higher in the younger population in recent years (Mazurek et al., 2024). GC easily leads to cancer cachexia with poor outcomes (Li et al., 2026). The treatment strategies and approaches of GC mainly include surgery, systemic chemotherapy and radiotherapy (Joshi and Badgwell, 2021; Alsina et al., 2022), which also cause side effects that greatly reduce the quality of life and the prognosis of patients. Although immunotherapy and targeted therapy, which target PD-1 inhibition, HER2-positive patients and angiogenesis, improve the treatment efficacy (Luo et al., 2025), the current treatment strategies and approaches for GC still do not provide satisfactory outcomes.

Programmed cell death (PCD), which includes apoptosis, necroptosis, pyroptosis, autophagy and ferroptosis, among others, is a common life phenomenon and is necessary for maintaining tissue function and morphology. In a healthy adult, approximately 50–100 billion cells die every day, which is equivalent to 0.4%–0.5% of all cells in the body (Gadiyar et al., 2020). Importantly, targeting the induction of PCD is the main strategy for anti-cancer drug development and therapy, including GC (Battaglia et al., 2020; Flores-Romero et al., 2020; Noguchi et al., 2020). Apoptosis is the most-known type of PCD that promotes effective elimination of cancer cells (Carneiro and El-Deiry, 2020). Autophagy is a process of cell self-phagocytosis and plays a bidirectional regulatory role in cancer growth (Chung et al., 2020; Cocco et al., 2020). Necroptosis is inflammation-related cell injury and death (Wang S. et al., 2024). Ferroptosis is a new type of PCD, which is different from apoptosis, necroptosis and autophagy, and it is characterized by lipid peroxidation and iron accumulation (Tang et al., 2019; Su et al., 2020). Zhu et al. found that tumor cells accelerated malignant a proliferation and metastasis through the activation of the PI3K/Akt and MAPKs signaling pathways (Zhu et al., 2019), which are closely associated with PCD. p53 acetylation at several C-terminal Lys residues is mainly mediated by p300 and its family member CBP acetyltransferases. p300 acetylates p53 at the amino acid resident of K370 and histones, which remodels the chromatin structure, and it cooperatively activates the transcription of target genes such as p21, ultimately regulating cellular physiological processes such as cell-cycle arrest and apoptosis (Heo et al., 2024). Thus, the promotion of PCD is still the main strategy for cancer treatment in western medicine (Tang et al., 2020).

Apart from western modern medicine, traditional Chinese medicine provides an alternative approach for GC treatment. The rhizome of *Anemarrhena asphodeloides* Bunge, which is named ‘Zhi-mu’ (知母) in Chinese, has been used as a tonic agent in various ethnomedicinal systems in East Asia, especially in China for hundreds of years (Lin et al., 2020). *A*. *asphodeloides* Bunge is widely used for treating arthralgia, hematochezia, tidal fever, night sweats, cough, dry mouth and tongue, and hemoptysis, among others (Liu C. et al., 2023). According to our previous studies, timosaponin AIII (Timo AIII) is an important active component isolated from *A*. *asphodeloides* Bunge and exerts multiple pharmacological activities, including anti-cancer, anti-neuronal disorders, anti-inflammation and anti-coagulant, among others (Lin et al., 2020; Zhou et al., 2020b). Timo AIII was proposed as a promising anti-cancer drug candidate that suppressed cancer progression in micromole grade concentration with low toxicity to normal cells (King et al., 2009; Zhou et al., 2020b; Liu Z. et al., 2023). The anti-cancer effect of Timo AIII is co-related with its anti-metastasis, anti-resistance, cytotoxicity, pro-apoptosis, and induction of cell-cycle arrest, reactive oxidant species (ROS), endoplasmic reticulum (ER) stress and mitochondria dysfunction. The main pharmacological targets of Timo AIII include vascular endothelial growth factor receptor (VEGFR), X-linked inhibitor of apoptosis protein (XIAP), B-cell-specific Moloney murine leukemia virus integration site 1 (BMI1), thromboxane (Tx) A2 receptor, mammalian target of rapamycin (mTOR), nuclear transcription factor-κB (NF-κB), cyclooxygenase-2 (COX-2), matrix metallopeptidases (MMPs) and acetylcholinesterase (AChE) (Lin et al., 2020). Additionally, zebrafish are considered a powerful vertebrate model for studying metastatic tumor *in vivo*, because they are similar to human tumors at the histological and cellular levels (Astell and Sieger, 2020; Raby et al., 2020). Timo AIII presented anti-angiogenesis effects, and its underlying mechanisms were involved in the suppression of VEGF/PI3K/Akt/MAPK signaling in human umbilical vein endothelial cell (HUVECs) and zebrafish (Zhou et al., 2020b). However, whether Timo AIII could directly inhibit GC progression is unknown. In this study, we explore the potential anti-GC effect of Timo AIII and its underlying mechanisms in zebrafish and human GC cell BGC-823.

## 2 Materials and methods

### 2.1 Ethics statement

Zebrafish experiments were conducted in accordance with the ethical guidelines of Longhua Hospital, Shanghai University of Traditional Chinese Medicine. All experimental protocols were approved by Longhua Hospital, Animal Ethics Committee of Shanghai University of Traditional Chinese Medicine.

### 2.2 Chemicals and reagents

Timosaponin AIII (Timo AIII, purity ≥98.0% by HPLC) was purchased from Chengdu Must Bio-Technology Co., Ltd (Chengdu, Sichuan, China). CellTracker™ CM-Dil (catalog no. C7000) was supplied by Thermo Fisher Scientific (MA, United States). LY294002 (catalog no. S1737), wortmannin (catalog no. S1952), SP600125 (JNKi, catalog no. S1876) and PD032591 (MEKi, catalog no. SD5924) were obtained from Beyotime Technology (Shanghai, China). Akt inhibitor IV (Akti, catalog no. 124015) was obtained from Calbiochem (Darmstadt, Germany). Z-VAD-(OME)-FMK (Z-VAD, catalog no. 14463), ferrostatin-1 (catalog no. 17729), necrostatin-1 (catalog no. 11658) and deferoxamine (catalog no. 14595) were obtained from CAYMAN Chemical company. N-acetyl cysteine (NAC, catalog no. A9165) was obtained from Sigma. The chemicals were dissolved in dimethyl sulfoxide (DMSO), PBS, or Milli-Q grated H_2_O according to their solubility.

### 2.3 Zebrafish maintenance and collection of embryos

The wild-type (WT) zebrafish line Tübingen (TU) was imported from China Zebrafish Center (Wuhan, China) and maintained in the Zebrafish Experimental Center, Longhua Hospital, Shanghai University of Traditional Chinese Medicine. Fish were maintained according to the fourth edition of The Zebrafish Book: A guide for the laboratory use of zebrafish *Danio (Brachydanio) rerio* by Monte Westerfield, Institute of Neuroscience, University of Oregon. In brief, zebrafish embryos were kept separately under a 14-h light/10-h dark cycle and fed brine shrimp twice a day. Zebrafish embryos were generated by natural pair-wise mating and incubated at 28.5 °C in the embryo medium (5 mM NaCl, 0.17 mM KCl, 0.33 mM CaCl_2_ and 0.33 mM MgSO_4_). After 48 h of incubation, embryos with healthy development were selected for later use. The zebrafish embryos were anaesthetized by MS-222 before microinjection or sacrifice.

### 2.4 Inoculation of BGC-823 cells to zebrafish embryos

At two days post-fertilization (dpf), zebrafish embryos with healthy development were selected, and cancer cell inoculation was performed according to our previous study (Shi et al., 2021). The BGC-823 cells were stained with 1 µM CM-Dil at 37 °C for 30 min and then washed thrice with PBS and suspended in PBS with the density of 3 × 10^6^ cells/ml. Zebrafish were microinjected with 10nl suspended BGC-823 cells (300 cells/per fish) in the yolk sac using the microinjection system (IM-31, NARISHIGE, Japan), and zebrafish that were injected with PBS served as the sham control. Zebrafish embryos were distributed in a 12-well plastic plate (n = 10) and treated with or without Timo AIII (1 µM) for 48 h. The stereoscopic fluorescence microscope (SMZ18, Nikon, Japan) was used for the observation of tumor growth and photography. The mean fluorescence intensity of the injection site was measured by NIS-Elements BR Analysis Software (version 4.30.00) in each zebrafish. Zebrafish in the control and PBS-injected groups were treated with DMSO (0.1%) as the vehicle controls. Ten zebrafish embryos were analyzed in each group, and this experiment was replicated thrice independently.

### 2.5 Cell viability assay

Human GC cell line BGC-823 was purchased from FUHENG Biology (Shanghai, China) along with the provided SRT genotyping report (Supplementary Material) and cultured in Dulbecco’s Modified Eagle’s Medium (DMEM) supplemented with 10% Fetal bovine serum (FBS) and 1% penicillin/streptomycin (PS) at 37 °C in a humidified atmosphere of 5% CO_2_ in air. BGC-823 cells were seeded at the density of 0.125 × 10^4^, 0. 25 × 10^4^, 0.5 × 10^4^, or 1.0 × 10^4^ cells/well in a 96-well plate and incubated for 24 h. Then, the BGC-823 cells were treated with various concentrations of Timo AIII (0.5, 1, 2 and 4 μM) for 24 h. The BGC-823 cells were treated with DMSO (0.1%) as the vehicle control. The cell viability of BGC-823 cells was tested by the thiazolyl blue tetrazolium (MTT) assay. After 24 h, the supernatant culture medium in the 96-well plate was discarded, and 100 µl of 1 mg/ml MTT was added and further cultured for 4 h in a 37 °C incubator. Then, the MTT solution was removed and 100 µl DMSO was added to each well. The absorbance of 490 nm was detected by multiple plate reader (infinite M200 PRO, Tecan). Cell viability was expressed as the percentage of the vehicle control.

For the evaluation of the effect of the inhibitions of the PI3K/Akt/MAPK, apoptosis, autophagy, ferroptosis and necroptosis signaling on the anti-GC effect of Timo AIII, various concentrations of autophagy inhibitor 3-MA (1, 3 and 10 μM), ferroptosis inhibitors ferrostatin-1 (2.5, 5 and 10 μM) and deferoxamine (5, 10 and 20 μM), apoptosis inhibitor Z-VAD (5, 10 and 20 μM), necroptosis inhibitor necrostatin-1 (10, 20 and 40 μM), ROS inhibitor NAC (0.1, 0.3 and 1 μM), PI3K inhibitors LY294002 (0.5, 1 and 2 μM) and wortmannin (0.25, 0.5 and 1 μM), Akt inhibitor IV Akti (0.1, 0.3 and 1 μM), MEK inhibitor MEKi (0.1, 0.3 and 1 μM), and JNK inhibitor JNKi (0.1, 0.3 and 1 μM) were co-treated with Timo AIII (2 μM) for 24 h in BGC-823 cells. The cell viability of BGC-823 was tested by the MTT assay as described above.

### 2.6 Real-time cell proliferation analysis by RTCA

The real-time proliferation of BGC-823 cells was monitored by a real-time cell analysis (RTCA) system named xCELLigence (ACEABIO S16, Hangzhou, China). The BGC-823 cells were seeded at a density of 5 × 10^3^ cells/well in a specified 16-well plate with treatments of various concentrations of Timo AIII (1, 2 and 4 μM) for 96 h. The BGC-823 cells treated with 0.1% DMSO served as the vehicle control. The BGC-823 cells were settled for 30 min before being placed in an xCELLigence instrument at 37 °C in a 95% humidified incubator with 5% CO_2_ in air. The cell independence, which is expressed as the cell index and presented the number of cells, was recorded at 1-h intervals.

### 2.7 Wound healing assay

The wound healing assay was used for the analysis of cell migration. The BGC-823 cells were seeded at a density of 5 × 10^4^ cells/well in a 12-well plastic cell culture plate and cultured to 100% confluence. The monolayer cells were wounded by 1-ml pipette tips to produce ‘cross scratches’ and washed with PBS to remove the non-adherent cells. Then, the BGC-823 cells were treated with various concentrations of Timo AIII (0.5, 1 and 2 μM) for 24 h. The BGC-823 cells treated with 0.1% DMSO served as the vehicle control. The cell morphology of each group was recorded by imaging after drug treatment for 0, 6, 12 and 24 h by an inverted microscope (Ti-U, Nikon, Japan) with ×4 magnification. The migration distance was measured on the field of cross scratches by ImageJ software (version 1.49 V).

### 2.8 Flow cytometry analysis

The flow cytometry analysis was used for the detection of cell cycle and cell apoptosis in BGC-823 cells. For the detection of cell apoptosis, BGC-823 cells were seeded at a density of 5 × 10^5^ cells/well in a specified 6-well plate and cultured to 70%–80% confluence in a CO_2_ incubator (37 °C and 5% CO_2_) for 24 h. The cells were treated with various concentrations of Timo AIII (1, 2 and 4 μM) and further incubated for 24 h. The cell treated with 0.1% DMSO served as the vehicle control. The cells were collected using EDTA-free trypsin digestion and followed by centrifuge at 4 °C. The cells were re-suspended with phosphate buffer, and Annexin V-FITC and propidium iodide (PI) were added for apoptosis cell staining according to the manufacturer’s instruction (Beyotime Biotechnology, Shanghai, China). Finally, the fluorescence intensity at the excitation wavelength of 488 nm and the emission wavelength of 530 nm was detected by flow cytometry (CytoFLEX, Beckman). For the detection of cell cycle, the BGC-823 cells were re-suspended in PBS, followed by the addition of ethanol to the final concentration of 70% (V/V) after Timo AIII drug treatment. After fixing overnight at 4 °C, BGC-823 cells were washed thrice with PBS, and the intracellular RNA was digested by RNase for 30 min at 37 °C. Then, the BGC-823 cells were stained with PI for 30 min in the dark at room temperature. The intracellular DNA content throughout the cell cycle was examined according to the manual of the cell-cycle analysis kit (Beyotime Biotechnology, Shanghai, China) using a flow cytometer (CytoFLEX, Beckman). The cell population was further calculated by FlowJo software (version 7.6.1).

### 2.9 Bioinformatics analysis of the central targets related to PCD

The potential targets related to PCD, including “apoptosis”, “autophagy”, “necroptosis” and “ferroptosis” were extracted from both the OMIM database (https://www.omim.org/) and the GeneCards database (https://www.genecards.org/) according to the previous studies (Stelzer et al., 2016; Cheng et al., 2021). A total of 359 targets of apoptosis, autophagy, necroptosis and ferroptosis are obtained using Venny 2.1 software. These 359 intersecting targets were imported to the String database (https://stringdb.org/) for protein–protein interaction (PPI) network construction (Chin et al., 2014), which was visualized by Cytoscape 3.10.1 software (Shannon et al., 2003). Metascape (http://metascape.org/) was used for gene function annotation analysis of the 359 targets. Biological processes with guiding significance were screened out, and Gene Ontology (GO) analysis and Kyoto Encyclopedia of Genes and Genomes (KEGG) pathway analysis were carried out (Kanehisa et al., 2019).

### 2.10 Real-time PCR analysis

BGC-823 cells were seeded in a 10-cm Petri dish with the cell density of 1 × 10^6^ cells/dish and cultured for 24 h. The cells were incubated with various concentrations (1, 2 and 4 μM) of Timo AIII for 24 h, followed by gene expression detection using real-time PCR analysis. Briefly, the total RNA was collected by RNAiso Plus (TaKaRa, Japan), and the RNA concentration was measured by a microplate reader (Infinite M200Pro NanoQuant, TECAN, Switzerland). The total RNA was transcribed to single-strand cDNA using a Transcriptor First-Strand cDNA Synthesis Kit (Roche, Manneheim, Germany). SYBGREEN PCR Master Mix (Roche, Manneheim, Germany) was used for real-time detection of the PCR product based on the Light Cycle 96 platform (LC96, Roche, Germany). The specific primers of the necroptosis- and ferroptosis-related genes are listed in Table 1. The relative mRNA expressions of the genes were normalized by the internal control GAPDH and calculated by the 2^−ΔΔCT^ method.

**TABLE 1:** Specific primers of related genes used in real-time PCR analysis.

Gene ID	Gene	Forward primer	Reverse primer
NM_001354932.2	RIPK1	5′-TGG​ACG​CCA​TTT​GGG​GAA​ATA-3′	5′-ATT​CTT​CTT​AGC​GGT​GCC​GT-3′
NM_006871.4	RIPK3	5′-CAT​GGA​GAA​CGG​CTC​CTT​GT-3′	5′- GGT​TCT​GGT​CGT​GCA​GGT​AA-3′
NM_001142497.3	MLKL	5′-TTC​CCT​CAG​GTA​GGG​ATC​GG-3′	5′-TGC​ACT​CTG​CTG​ACT​GTA​CC-3′
NM_001711.6	BGN	5′- GGT​GGC​TAG​GTC​TCC​CCT​TA-3′	5′-CAC​GTT​GCA​CGG​TGT​TTC​TT-3′
NM_014331.4	SLC7A11	5′-CGC​TGA​GAG​AGA​CAG​TCT​GA-3′	5′-TGG​TGG​ACA​CAA​CAG​GCT​TT-3′
NM_000963.4	PTGS2	5′-GTT​CCA​CCC​GCA​GTA​CAG​AA-3′	5′-AGG​GCT​TCA​GCA​TAA​AGC​GT-3′
NM_001256799.3	GAPDH	5′-ATG​GCA​AAT​TCC​ATG​GCA​CC-3′	5′-GAC​TCC​ACG​ACG​TAC​TCA​GC-3′

### 2.11 Western blotting analysis

BGC-823 cells were seeded in a 10-cm Petri dish with the cell density of 1 × 10^6^ cells/dish and cultured for 24 h. The cells were incubated with various concentrations (1, 2 and 4 μM) of Timo AIII for 24 h, followed by protein expression assay using Western blotting analysis. Briefly, BGC-823 cells were washed thrice with ice-cold PBS and lysed in RIPA buffer. Cell lysates were centrifuged at 12,000 × g for 15 min at 4 °C. The protein concentration was measured using a BCA assay kit (Pierce, Rockford, IL, United States). Then, protein was denatured in loading buffer, and 30 μg of the total protein from each sample was separated by SDS-PAGE and transferred to a polyvinylidene fluoride membrane (0.45 μm), which was then blocked with 5% BSA. Immunoblots were incubated with primary antibodies, including antibodies against phospho-Akt (CST, catalog no. #2965s), Akt (CST, catalog no. #4685s), phospho-ERK1/2 (CST, catalog no. 4370S), ERK1/2 (CST, catalog no. 4695s), phospho-MEK1/2 (CST, catalog no. #9154S), MEK1/2 (CST, catalog no. 9122S), Bcl2 (CST, catalog no. 2870s), Bax (CST, catalog no. 2772s), Cleaved-Caspase3 (CST, catalog no. #9665s), LC3A/B (CST, catalog no. 12741T), Atg5 (CST, catalog no. 12994T), Atg12 (CST, catalog no. 4180T), Atg16 (CST, catalog no. 8089T), GPX4 (abcam, catalog no. ab125066), Acetyl-p53-K370 (ABclonal, catalog no. A19836), p53 (ABclonal, catalog no. A25915), p300 (Santa Cruz Biotechnology, catalog no. sc-48343) and GAPDH (CST, catalog no. 5174S) with 1:1,000 dilution at 4 °C overnight. After washing thrice with PBST, the immunoblots were incubated with horseradish peroxidase-conjugated secondary antibody (1:2,000, CST) for 2 h at room temperature. After the final wash, the immunoblots were visualized using an enhanced ECL system (BeyoECL moon, Beyotime, Shanghai, China) and imaged on an imaging system (GE AM600, Fairfield, CT, United States). The mean intensity of each protein band was analyzed by ImageJ software (version 1.49V). The protein expression levels were normalized to the control group.

### 2.12 Intracellular iron and MDA assay

BGC-823 cells were seeded in a 60-mm Petri dish with the density of 5 × 10^5^ cells/dish and cultured for 24 h. Then, cells were treated with various concentrations (1, 2 and 4 μM) of Timo AIII for 24 h. The intracellular iron and intracellular malondialdehyde (MDA) were detected by an Tissue Iron Assay Kit (Nanjing Jiancheng Bioengineering Institute, Nanjing, China) and a Lipid Peroxidation MDA Assay Kit (Beyotime, Shanghai, China) according to the manufacturer’s instructions, respectively. In the meantime, the protein concentration was detected using the BCA kit (Epizyme, Shanghai, China). Intracellular iron and MDA were normalized to the protein concentration. Finally, the results were normalized to the folds of the control group.

### 2.13 Molecular docking

Molecular docking analysis was performed to predict the binding affinity of Timo AIII with the p300 protein. The simulations were conducted using AutoDock software (version: AutoDock Tools-1.5.7), and the interactions between the compound and the key residues of p300 were visualized with PyMol software (version: PyMol-3.1), following the methodology described in a previous study (Kitchen et al., 2004). The procedure was as follows: first, the crystal structure of human p300 (PDB ID: 6V8K) was retrieved from the Protein Data Bank (https://www.rcsb.org/). The water molecules were removed, and the hydrogen atoms were added to the structure. The structure of Timo AIII (CID: 71306914) was obtained from the PubChem database (https://pubchem.ncbi.nlm.nih.gov/), and its three-dimensional geometry was optimized using Open Babel-3.1.1 (Zhang et al., 2021). Subsequently, the Timo AIII molecule was converted from the SDF to the PDB format. The hydrogen atoms were added into Timo AIII’s structure, followed by the detection of the root of the ligand and the definition of rotatable bonds, and finally the structure file was saved in the pdbqt format. Next, a docking grid box was set around the active sites of p300 using AutoDock Tools (Ping et al., 2022). The center of the grid box was set at coordinates (x, y, z) = (10, 20, 30), with the dimensions of 20 A × 20 A × 20 A. Docking binding energy calculations were performed using the AutoDock Vina-1.5.7 command line. The results were analyzed in PyMOL, with a focus on evaluating the binding energy, where more negative values <7.0 kcal/mol indicate stronger binding (Zhang et al., 2021) and ensuring proper conformational fitting of the ligand within the active site.

### 2.14 Data analysis

Data were presented as the mean ± S.E.M. from at least three independent experiments. The Student’s t-test or analysis of variance (ANOVA) was used to perform statistical evaluations of the difference between two groups, and *p* < 0.05 was considered significant difference.

## 3 Results

### 3.1 Timo AIII inhibited human gastric cancer cell growth in zebrafish

**FIGURE 1:**
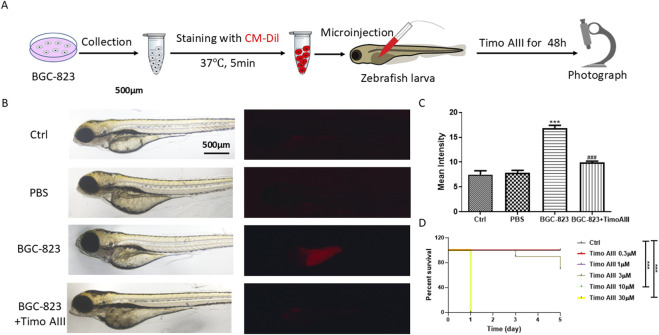
Effect of Timo AIII on the gastric cancer cell growth in zebrafish. **(A)** Experimental flow. **(B,C)** Two days post-fertilization (dpf) WT zebrafish embryos were injected with BGC-823 cells (300 cells/per fish) and then treated with Timo AIII (1 µM) for 48 h. Zebrafish embryos injected with PBS served as the vehicle control. The mean fluorescence intensity of the injection site was calculated by ImageJ software (version: 1.53a). n = 5. **(D)** One dpf WT zebrafish embryos were treated with various concentrations of Timo AIII (0.3, 1, 3, 10 and 30 µM) for five days, and the survival rate was recorded daily. n = 5. Data were presented as mean ± S.E.M. ***p < 0.001 versus the control group or the PBS-injected group. ###*p* < 0.001 versus the BGC-823 cells-inoculated group.

To detect the effect of Timo AIII on human GC cell proliferation *in vivo*, the WT zebrafish inoculated with human GC cells BGC-823, which were pre-labeled by staining with fluorescent DNA dye CM-Dil, was established, followed by the treatment with Timo AIII (Figure 1A). The inoculation of BGC-823 cells markedly increased the mean fluorescence intensity in the microinjection site than that in the PBS-injected group. Compared with the BGC-823 cells-inoculated group, the mean fluorescence intensity of the cells significantly decreased in the Timo AIII (1 µM)-treated group (Figures 1B, C). Moreover, Timo AIII did not decrease the survival rate within the concentration range of ≤ 3 µM in zebrafish (Figure 1D). These results reveal that Timo AIII inhibited human GC cell growth in zebrafish without toxicity.

### 3.2 Timo AIII inhibited human GC cell proliferation and migration

**FIGURE 2:**
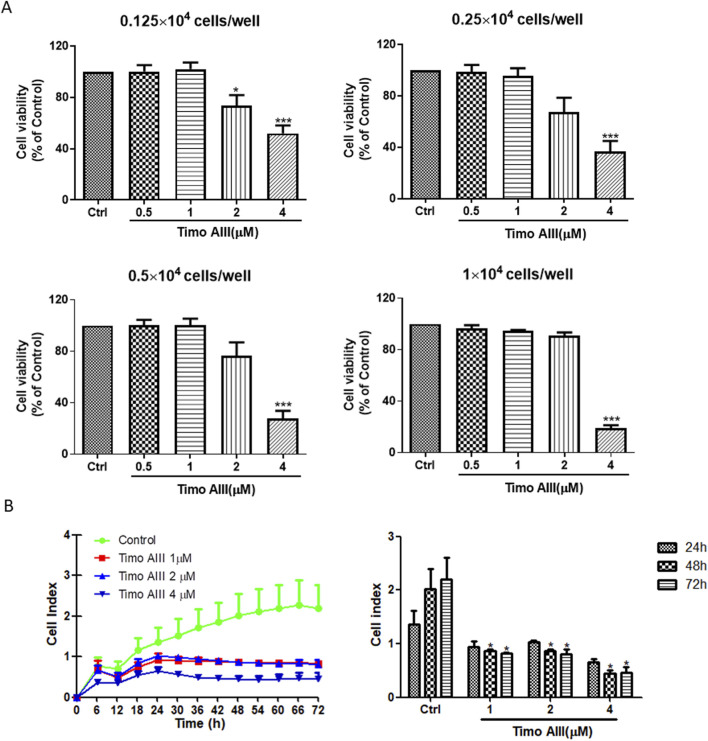
Effect of Timo AIII on the cell viability and proliferation in BGC-823 cells. **(A)** BGC-823 cells, which were seeded with the indicated density in a 96-well cell-culture plate, were treated with various concentrations of Timo AIII (0.5, 1, 2 and 4 μM) for 24 h, and the cell viability was tested by the MTT assay. n = 3. **(B)** Cell impedance, which was expressed as the cell index and measured by the xCELLigence RTCA system, was used to evaluate the real-time proliferation of BGC-823 cells. The BGC-823 cells were treated with various concentrations of Timo AIII (1, 2, and 4 μM) for 72 h, and the cell impedance was detected at 1-h intervals. n = 3. Data were presented as mean ± S.E.M. *p < 0.05 and ***p < 0.001 versus the control group.

**FIGURE 3:**
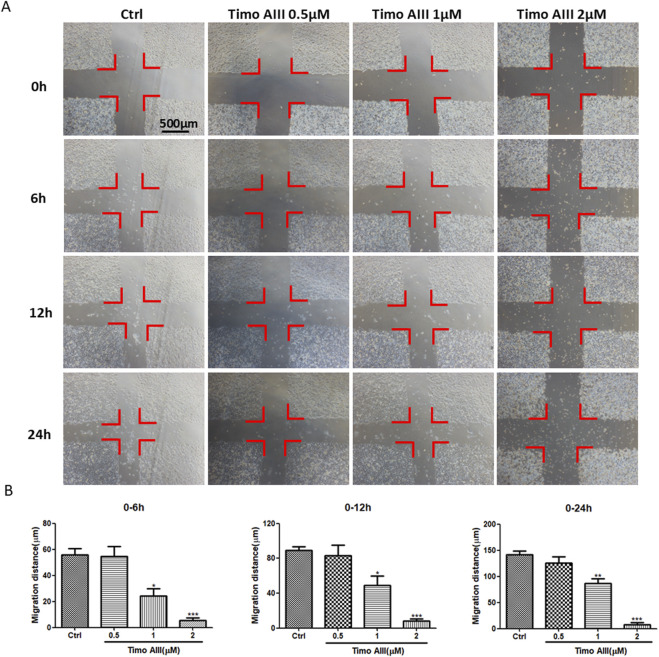
Effect of Timo AIII on the migration of BGC-823 cells. **(A,B)** After the BGC-823 cells were cultured to a monolayer, the cells were scratched by a 1-ml plastic tip and treated with Timo AIII (0.5, 1 and 2 μM) for 24 h. Then, the cells were photographed by an inverted microscope with a 4 × objective. The migration distance of BGC-823 cells was measured by ImageJ software. n = 3. Data were presented as mean ± S.E.M. *p < 0.05, **p < 0.01, and ***p < 0.001 versus the control group.

Cell proliferation and migration play important roles in the process of tumor development and progression, particularly metastasis (Chen et al., 2022). We evaluated the effect of Timo AIII on the cell viability of BGC-823 cells with various cell-seeding densities since the cell density affects the drug response in the cell study (Greene et al., 2016). We found that Timo AIII concentration-dependently decreased the cell viability of BGC-823 cells (Figure 2A). The cell viability of BGC-823 cells was significantly decreased in the Timo AIII (2 and 4 µM)-treated groups, while Timo AIII did not present significant cytotoxicity within the concentration range of ≤1 μM (Figure 2A). As seen in the results from the xCELLigence real-time cell analysis (RTCA) system, Timo AIII markedly suppressed the proliferation of BGC-823 cells in both concentration- and time-dependent manners (Figure 2B). Moreover, we used a wound-healing assay to measure the effect of Timo AIII on the migration of BGC-823 cells, and the migration distance reduced concentration- and time-dependent in the Timo AIII-treated BGC-823 cells (Figures 3A,B). These results reveal that Timo AIII significantly decreased the cell viability and inhibited both the cell proliferation and migration in human GC.

### 3.3 Timo AIII blocked human GC cell-cycle progression and promoted cell apoptosis

**FIGURE 4:**
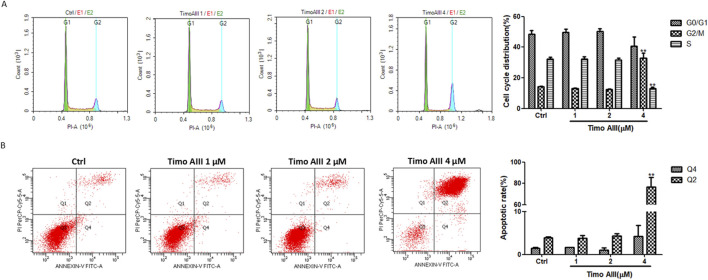
Effects of Timo AIII on the cell-cycle progression and apoptosis of BGC-823 cells. **(A)** BGC-823 cells were treated with various concentrations of Timo AIII (1, 2 and 4 μM) for 24 h. The cell-cycle analysis was performed through flow cytometry after PI-staining. The representative images presented the cell-cycle data analysis using FlowJo software (version 7.6.1). n = 4. **(B)** Cell apoptosis analysis was carried out through flow cytometry after Annexin V-FITC and PI double-staining. n = 4. Data were presented as mean ± S.E.M. **p < 0.01 versus the control group.

Induction of cell-cycle arrest is one of key action mechanisms of the current cancer treatment drugs. For example, trilaciclib and ALRN-6924 cause a temporary cell-cycle arrest in small-cell lung cancer treatment (Recasens and Munoz, 2019; Falandry et al., 2023). To explore whether Timo AIII blocked the cell-cycle progression of BGC-823 cells, we measured the cell-cycle progression by detecting intracellular DNA content using flow cytometry after PI-staining in BGC-823 cells, along with the analysis of the cell-cycle distribution. We found that Timo AIII (4 µM) significantly decreased the number of S-phase cells and increased the number of G2/M phase cells (Figure 4A). Additionally, acceleration of cell apoptosis is an effective strategy for the induction of cancer cell death (Li et al., 2024a). We detected the cell apoptosis rate using Annexin V-FITC and PI double-staining by flow cytometry analysis. Based on the data shown in Figure 4B, Timo AIII (4 µM) increased cell distribution in the Q2 region (Annexin V-FITC and PI double-positive), which presented the later apoptosis cells. These results reveal that Timo AIII blocked the cell-cycle progression and enhanced cell apoptosis in human GC.

### 3.4 Inhibition of PCD signaling reduced the anti-GC effect of Timo AIII

**FIGURE 5:**
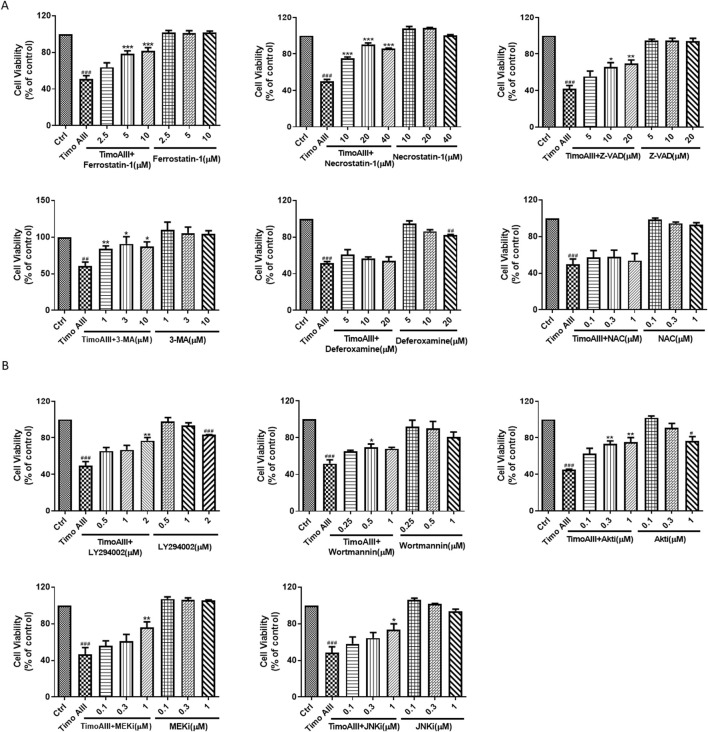
Effect of the inhibition of PI3K/Akt/MAPK, apoptosis, autophagy, ferroptosis and necroptosis signaling on the anti-GC activity of Timo AIII. **(A)** Various concentrations of the autophagy inhibitor 3-MA (1, 3 and 10 μM), ferroptosis inhibitors ferrostatin-1 (2.5, 5 and 10 μM) and deferoxamine (5, 10 and 20 μM), apoptosis inhibitor Z-VAD (5, 10 and 20 μM), necroptosis inhibitor necrostatin-1 (10, 20 and 40 μM), and ROS inhibitor NAC (0.1, 0.3 and 1 μM) were co-treated with Timo AIII (2 μM) in BGC-823 cells for 24 h. n = 3. **(B)** Various concentrations of Akt inhibitor IV Akti (0.1, 0.3 and 1 μM), JNK inhibitor JNKi (0.1, 0.3 and 1 μM), MEK inhibitor MEKi (0.1, 0.3 and 1 μM), and PI3K inhibitors LY294002 (0.5, 1 and 2 μM) and wortmannin (0.25, 0.5 and 1 μM) were co-treated with Timo AIII (2 μM) in BGC-823 cells for 24 h. n = 3. The cell viability was measured by MTT assay. The cell viability was presented as the percentage of the control group. Data were presented as mean ± S.E.M. ##*p* < 0.01 and ###*p* < 0.001 versus the control group; *p < 0.05, **p < 0.01, and ***p < 0.001 versus the Timo AIII-treated group.

To further confirm the underlying mechanisms of the anti-GC effect of Timo AIII, we pharmacologically blocked PCD, including apoptosis, autophagy, ferroptosis and necroptosis by small molecular inhibitors. We found that the ferroptosis inhibitor (ferrostatin-1), necroptosis inhibitor (necrostatin-1), apoptosis inhibitor (Z-VAD) and autophagy inhibitor (3-MA) partially abolished the anti-cancer effect of Timo AIII in a concentration-dependent manner, while the iron chelator deferoxamine and ROS inhibitor N-acetyl cysteine (NAC) did not affect the anti-proliferation effect of Timo AIII in BGC-823 cells (Figure 5A). Both cell survival and PCD are regulated by PI3K/Akt/MAPKs signaling (Bie et al., 2020). We found that the PI3K inhibitors (LY294002 and wortmannin), Akt inhibitor IV (Akti), MEK inhibitor (MEKi) and JNK inhibitor (JNKi) concentration-dependently reduced the anti-GC effect of Timo AIII (Figure 5B). These results reveal that the underlying mechanisms of the anti-GC effect of Timo AIII were associated with the induction of PCD, along with the activation of PI3K/Akt/MAPKs signaling.

### 3.5 Timo AIII upregulated the markers of PCD in GC

**FIGURE 6:**
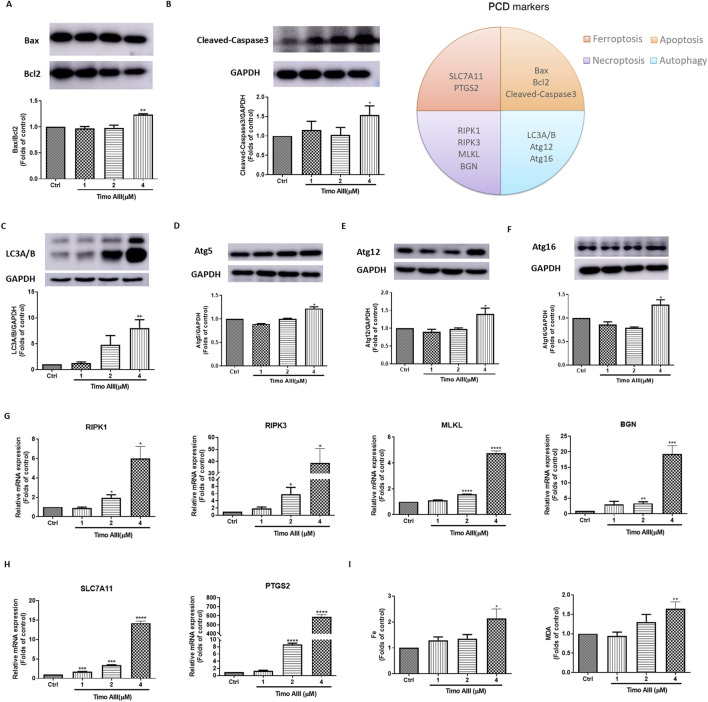
Effects of Timo AIII on the markers of apoptosis, autophagy, necroptosis and ferroptosis in BGC-823 cells. **(A–F)** BGC-823 cells were treated with various concentrations of Timo AIII (1, 2 and 4 μM) for 24 h. The total protein of Bax, Bcl2, Cleaved-Caspase3, LC3A/B, Atg5, Atg12 and Atg16 were detected by Western blotting analysis. n = 3. **(G–H)** mRNA expression levels of RIPK1, RIPK3, MLKL, BGN, SLC7A11 and PTGS2 were detected by real-time PCR. n = 3. **(I)** Intracellular iron and MDA were detected according to the manufacturers. n = 3. The results were presented as the folds of the control group. Data were presented as mean ± S.E.M. *p < 0.05, **p < 0.01, ***p < 0.001, and ****p < 0.0001 versus the control group.

PCD, including apoptosis, autophagy, necroptosis and ferroptosis, is involved in the process of death in various types of tumors (Wendlocha et al., 2024; Niu et al., 2025). We found that Timo AIII increased the apoptosis markers Bax/Bcl2 and Cleaved-Caspase3 (Figures 6A,B) and the autophagy marker LC3A/B (Figure 6C). Meanwhile, Malte et al. have shown that Atg5/12/ 16 and their complexes play independent and synergistic roles in typical autophagy (Karow et al., 2019). Timo AIII also elevated the protein levels of other autophagy markers, including Atg5, Atg12 and Atg16, in BGC-823 cells (Figures 6D–F). Consistently, Timo AIII remarkably elevated the mRNA expression of necroptosis markers, including RIPK1, RIPK3, MLKL and BGN (Figure 6G), and ferroptosis markers, including SLC7A11, PTGS2 (Figure 6H), intracellular lipid peroxidation (MDA) content, and intracellular iron content (Figure 6I), in BGC-823 cells. These results indicate that the anti-GC effect of Timo AIII was involved in PCD, including apoptosis, autophagy, necroptosis and ferroptosis.

### 3.6 p300/acetyl-p53 and Akt/MEK/ERK signaling were involved in Timo AIII-induced PCD in GC

**FIGURE 7:**
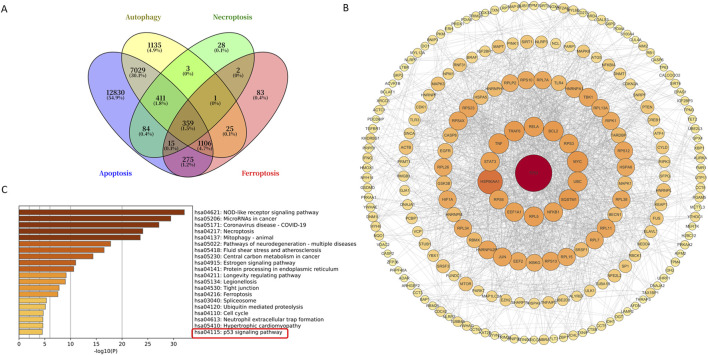
Bioinformatic analysis of genes associated with PCD. **(A)** Venn diagram. **(B)** PPI network diagram. **(C)** Bar plot of the KEGG pathway.

**FIGURE 8:**
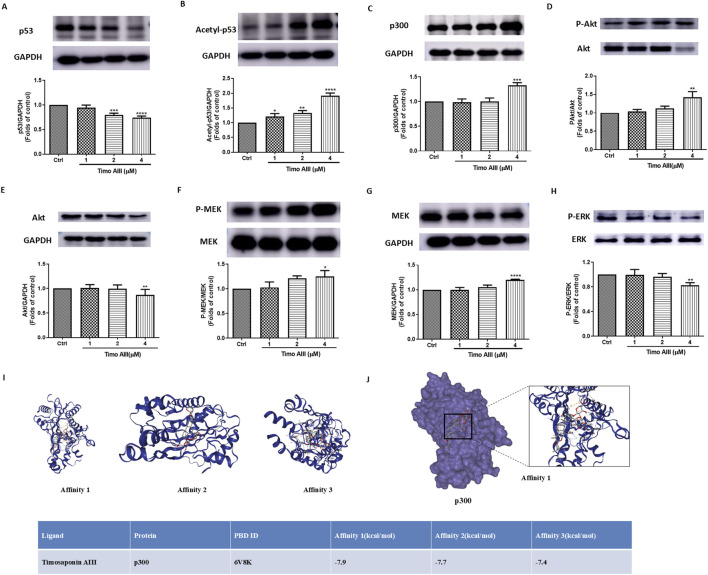
Effects of Timo AIII on proteins related to p53 and Akt/MEK/ERK signaling in BGC-823 cells **(A–H)**. The BGC-823 cells were treated with various concentrations of Timo AIII (1, 2 and 4 μM) for 24 h. The total protein of p53, Acetyl-p53 and p300 and phosphorylated protein of Akt, MEK and ERK were detected by Western blotting analysis GAPDH served as the internal control. n = 3. **(I,J)** *In-silico* binding of Timo AIII with p300 (PDB ID: 6V8K) was predicted by molecular docking using AutoDock software with visualization by PyMol software. The results were presented as the folds of the control group. Data were presented as mean ± S.E.M. *p < 0.05, **p < 0.01, ***p < 0.001, and ****p < 0.0001 versus the control group.

We extracted the genes that are associated with apoptosis, autophagy, necroptosis and ferroptosis from OMIM and GeneCards databases and found that there were 359 overlaid genes (Figure 7A). These genes were further analyzed by PPI network analysis and KEGG enrichment, and the tumor-suppressor protein p53 and its regulated signaling might play a central role across these four types of PCD (Figures 7B,C). We hypothesized that p53 might be the key target of Timo AIII, inducing these four types of PCD. p53 acetylation is important for its function, and the increased level of acetylized p53 enhanced its stability and anti-tumor ability (Wang D. et al., 2024). We found that Timo AIII slightly inhibited the protein expression of p53 while robustly elevating the acetylated p53 (K370) level (Figures 8A,B). Timo AIII enhanced the acetylation of p53 (K370) by upregulating the protein expression of p300 (Figure 8C). To predict whether Timo AIII directly binds to p300, we performed in-silico molecular docking using AutoDock software to assess the binding affinity between Timo AIII and p300. Molecular docking results reveal that Timo AIII interacts with p300 at three distinct sites, namely, one primary active domain (site one) and two regulatory domains (sites two and three), and the corresponding binding affinities were calculated to be −7.9, −7.7 and −7.4 kcal/mol, respectively (Figures 8I,J). These results indicate that in addition to increasing the intracellular p300 protein expression level, Timo AIII may directly bind to p300 to further enhance its activity. In addition, the Akt/MEK/ERK signaling pathways mediated multiple cellular homeostasis and the cell metabolism process (Gao et al., 2019; Ma et al., 2021). Timo AIII increased the MEK and phosphorylation of Akt and MEK and suppressed the Akt and phosphorylation of ERK (Figures 8D–H). These results indicate that Timo AIII promoted the p300-mediated acetylation of p53 and Akt/MEK/ERK signaling activation.

## 4 Discussion

GC is becoming one of the common causes of cancer death worldwide, accompanied by the increasing unhealthy lifestyles (Zheng et al., 2023). Timo AIII is a promising anti-cancer candidate that has demonstrated its effects in various types of cancers, except GC. In this study, we addressed the anti-GC effect of Timo AIII for the first time, and the underlying molecular mechanisms were partially illustrated.

Zebrafish is a classic model organism for the analysis of gene function and development and is also emerging as a human disease model in recent decades, and it is particularly suitable for high- or medium-throughput drug screening (Shimizu et al., 2023). In our laboratory, we established various zebrafish human disease models and accumulated significant experience for the evaluation of the active ingredients of traditional Chinese medicine and studying the underlying mechanism (Zhou et al., 2018; Zhou et al., 2019; Zhou et al., 2020a; Zhou et al., 2020b). We previously established a human breast cancer model in zebrafish by the inoculation of MDA-MB-231 or 4T1 cells in the yok sac of zebrafish embryo (Shi et al., 2021). In this study, the human GC model was successfully constructed by the inoculation of BGC-823 cells in zebrafish, and Timo AIII inhibited tumor growth with low toxicity (Figure 1), which provided the first evidence of the anti-GC effect of Timo AIII, along with its safety in the following drug development. Moreover, we found that Timo AIII presented anti-angiogenesis activity in both zebrafish embryos and HUVECs in our previous study (Zhou et al., 2020b). Therefore, Timo AIII might also inhibit the tumor angiogenesis in BGC-823-inoculated zebrafish embryo, leading to the suppression of the growth of GC. Cell proliferation and migration initiate cancer progression and metastasis, and inhibition of cancer cell proliferation and induction of cancer cell death are still the key mechanisms of first-line cancer treatment drugs, such as chemotherapy and radiotherapy (Ghiaseddin et al., 2020). In line with the result from the zebrafish model, Timo AIII significantly inhibited the proliferation of human GC cells at the grade of micromole concentration (Figure 2), accompanied with the reduction of GC cell migration (Figure 3). Moreover, cell cycle and cell apoptosis are involved in several biological processes, such as cell differentiation, proliferation, and removal of damaged cells (Shah et al., 2023). Induction of cell apoptosis or cell-cycle arrest is a classical strategy in the treatment of cancer (Ding et al., 2020). In this study, Timo AIII aggravated cell apoptosis and blocked the cell-cycle progression in GC cells (Figure 4). Inhibition of GC cell proliferation and migration by Timo AIII might be due to the promotion of cell apoptosis and cell-cycle arrest. These results prove that Timo AIII markedly inhibited GC progression both in BGC-823 cells *in vitro* and zebrafish embryos *in vivo*, which enhances its potency in the development of anti-GC drugs.

**FIGURE 9:**
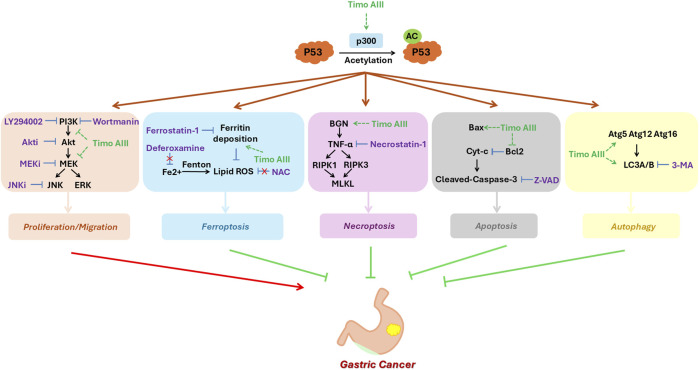
Schematic overview of the underlying action mechanisms involved in the anti-GC effect of Timo AIII.

Previous studies have demonstrated that Timo AIII could promote apoptosis, autophagy, cell-cycle arrest, and suppression of cell migration and invasion in various cancer types (Liu Z. et al., 2023). Interestingly, almost all the PCD inhibitors partially affect the anti-GC effect of Timo AIII without toxicity (Figure 5A). The promotion of PCD, including ferroptosis, necroptosis, apoptosis and autophagy, was involved in the anti-GC activity of Timo AIII, which is possible and consistent with the results of the previous study, although the induction of autophagy was highlighted (Gao et al., 2022). In addition, according to these results from PCD inhibitors (Figure 5A), we could confirm that autophagy, ferroptosis and necroptosis are the primary processes induced by Timo AIII, and these processes do not appear to be independent. However, excessive ROS-induced cell death might not be involved in the anti-GC effect of Timo AIII because its ROS scavenger NAC did not affect the anti-GC activity of Timo AIII (Figure 5A). In addition, PI3K/Akt/MAPKs signaling fundamentally regulates tumor proliferation, migration, metastasis and PCD (Wang D. et al., 2024), and the regulation of this signaling cascade is also involved in the anti-GC effect of Timo AIII (Figure 5B). In line with these results, Timo AIII increased the markers of PCD (Figure 6). According to the KEGG enrichment and PPI network analysis of the overlaid genes from all kinds of PCD, the tumor-suppressor p53 and its related signaling might play a central role (Figure 7), and we verified that Timo AIII did regulate the protein level and acetylation of p53 and the p53 acetyltransferase p300 (Figures 8A–C) in human GC. Timo AIII increased the acetylation level of p53, although the protein level of p53 decreased slightly, which might be due to the cell detrimental effect of Timo AIII. Furthermore, molecular docking results indicated that Timo AIII exhibits high binding affinity for the active pocket of p300 (Figures 8I,J). The K370 site on p53 is a crucial lysine residue in its C-terminal regulatory region, which mainly dynamically regulates the function of p53 (Li et al., 2024b) an is acetylated by p300 (Chen et al., 2024). These bioinformatics analysis strategy and further experimental verification reveal the connection of all types of PCD, and targeting the regulation of multiple types of PCD is possible in anti-cancer drug development. p53 is a strong regulator of cell proliferation, cell cycle and apoptosis (Wang et al., 2023), which might be the underlying mechanism of Timo AIII inhibiting GC cell proliferation and apoptosis and inducing cell-cycle arrest (Figures 2–4). Furthermore, in human GC, Timo AIII promoted the activation of Akt/MEK/ERK signaling (Figures 8D–H), which is in agreement with the results that the inhibition of this signaling pathway partially blocked the anti-GC effect of Timo AIII (Figure 5B). Although Akt is generally regarded as a pro-survival kinase, its excessive or dysregulated activation can exert pro-apoptotic effects in a cell-dependent manner, especially in cancer cells with aberrant pathway homeostasis (Los et al., 2009). Liao et al. revealed an antagonistic relationship between Akt and ERK in MC-LR-treated GC malignancy (Liao et al., 2023). All the mechanistic study results further confirmed the anti-GC effect of Timo AIII and its potential mechanisms (Figure 9). Our study provides evidence for the anti-GC effect of Timo AIII. However, further verification is still needed in rodent models in the future. In 2022, it was highlighted that the BGC-823 cell is one of the most frequently misidentified cell lines, with HeLa contamination being the primary cause, and this misidentification has led to widespread misleading results in gastric cancer research (Souren et al., 2022). We are also not confident regarding this matter, although the supplier (FUHENG Biology, Shanghai) proved that the cells used in this study were from a pure GC cell line with a SRT genotyping report (Supplementary Material) before. It is better to use other GC cell lines in future studies to provide more irrefutable evidence of Timo AIII’s anti-GC effect.

## 5 Conclusion

Timo AIII inhibited GC proliferation and migration by the induction of PCD, and the underlying mechanisms at least partially involved the regulation of p300/acetyl-p53 and Akt/MEK/ERK signaling. We analyzed the anti-GC effect and its underlying molecular mechanisms of Timo AIII for the first time, and Timo AIII might be a promising drug candidate for the treatment of GC.

## Data Availability

The raw data supporting the conclusions of this article will be made available by the authors, without undue reservation.
