# Supplementary material for: Timosaponin AIII inhibits gastric cancer growth by the promotion of programmed cell death via the activation of p300/acetyl-p53 and Akt/MEK/ERK signaling
Source: Front Pharmacol. 2026 Jul 20;17:1821689. doi: 10.3389/fphar.2026.1821689 (PMC13429718; doi:10.3389/fphar.2026.1821689)

细胞遗传质量鉴定检验报告

检品名称：细胞系

送检单位：富衡生物

检验类型：STR 基因型检验

发出时间：2016 年 8 月 23 号

遗传质量鉴定检验报告

样品编号：

表 1 样本编号

客户样本编号	公司编号
BGC-823	20160816-05

样品数量： 1

样品性状： 细胞系

检测项目： STR

送检单位： 富衡生物

检测方法： 用 Axygen 的基因组抽提试剂盒提取 DNA，采用 20- STR 扩增方案扩增，在 ABI 3730XL 型遗传分析仪上对 STR 位点和性别基因 Amelogenin 进行检测。

检验结果：

(一)检验基本情况

表 2：样本基因型检验结果

样本编号	多等位基因	匹配细胞系	细胞库	匹配说明
20160816-05	无	BGC823	CRC	基本匹配

- 多等位基因指三等位及以上基因现象。
- 本次检测各细胞分型结果良好。

(二)各样本描述

- 20160816-05：该株细胞 DNA 分型在细胞系检索中找到基本匹配的细胞系，CRC 数据

库显示细胞名为 **BGC823 等**，细胞号对应 **238 等**。本次检测在该细胞系中**没有发现多等位基因**。

备注：待测细胞系与收录于 ATCC, DSMZ, JCRB 和 RIKEN 数据库的细胞系 STR 数据进行比对，未收录于以上细胞库的细胞系将无法匹配。

(三)样本分型结果

表 3：细胞 20160816-05 的 STR 位点和 Amelogenin 位点的基因分型结果

Marker	样本				细胞库信息		
	**Allele1**	**Allele2**	**Allele3**	**Allele4**	**Allele1**	**Allele2**	**Allele3**
D5S818	11	12			11	12	
D13S317	12	13.3			12	13	
D7S820	8	12			8	12	
D16S539	9	10			9	10	
VWA	16	18			16	18	
TH01	7	7			7	7	
AMEL	X	X			X	X	
TPOX	8	12			8	12	
CSF1PO	9	10			9	10	
D12S391	20	25					
FGA	21	21					
D2S1338	17	17					
D21S11	27	28					
D18S51	16	16					
D8S1179	12	13					
D3S1358	15	18					
D6S1043	18	19					
PENTAE	7	17					
D19S433	12	13					
PENTAD	8	15					

其他说明:

(一)分型方案及位点分布:

附表：实验方案及位点

	**方案 1**	**方案 2**	**方案 3**	**方案 4**	**方案 5**	**方案 6**

1	TH01	AMEL	TPOX	D3S1358	PENTAE	D19S433
2	D12S391	D5S818	VWA	D13S317		PENTAD
3	D7S820	D2S1338	D8S1179	D6S1043		
4	CSF1PO	D21S11		D16S539		
5	FGA	D18S51				

(二)STR 数据库比对:

本公司采用 DSMZ tools 进行细胞系比对，其中包含来自于 ATCC, DSMZ, JCRB 和 RIKEN 数据库的 2455 个细胞系 STR 数据。如果待检测细胞未收录于以上细胞库或这是自行建立的新细胞系将无法进行比对，用户需根据细胞分型结果自行与其他数据库进行比对。

签发日期：2016 年 8 月 23 号

Sample File	Sample Name	Panel	OS	SQ
13_E02_XiBaoJianDing0816-0818.fsa	BGC-823	STR Profile 1-dup		

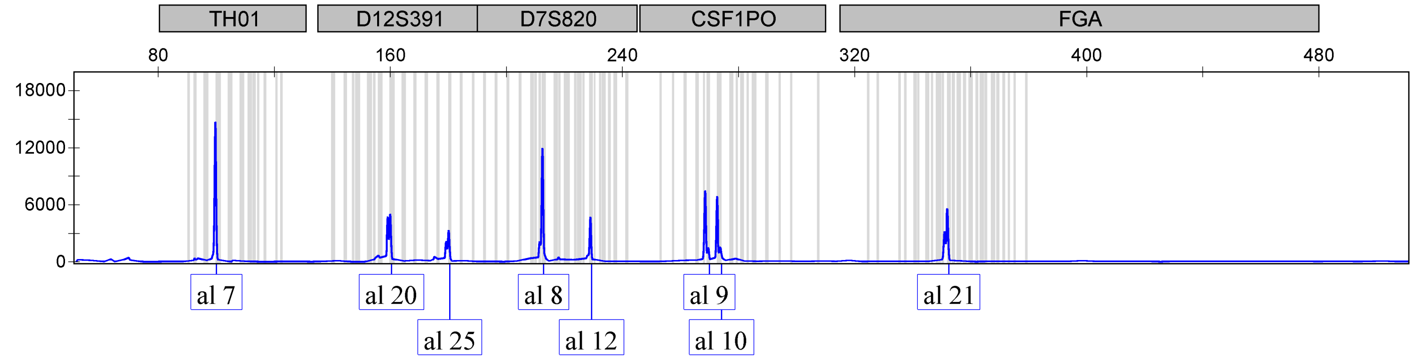

Sample File	Sample Name	Panel	OS	SQ
37_E05_XiBaoJianDing0816-0818.fsa	BGC-823	STR Profile 2-dup		

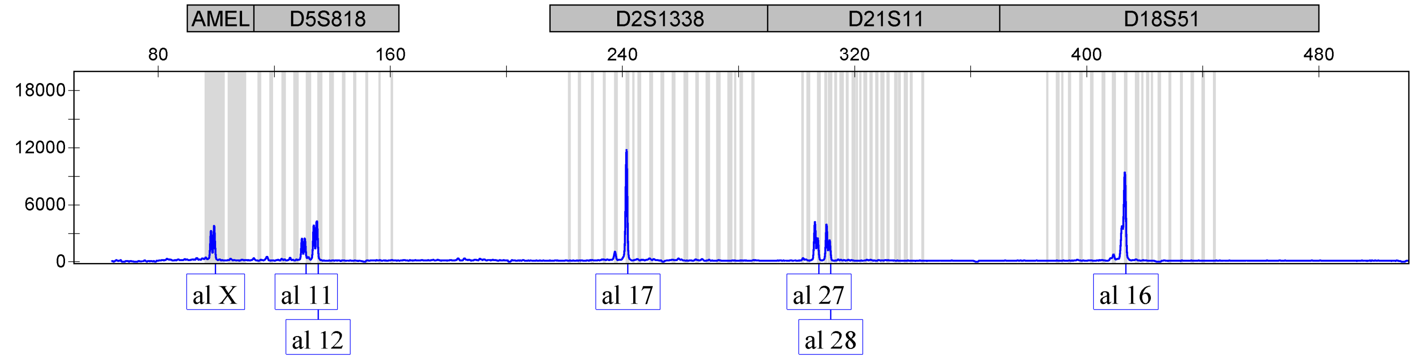

Sample File	Sample Name	Panel	OS	SQ
53_E07_XiBaoJianDing0816-0818.fsa	BGC-823	STR Profile 3-dup		

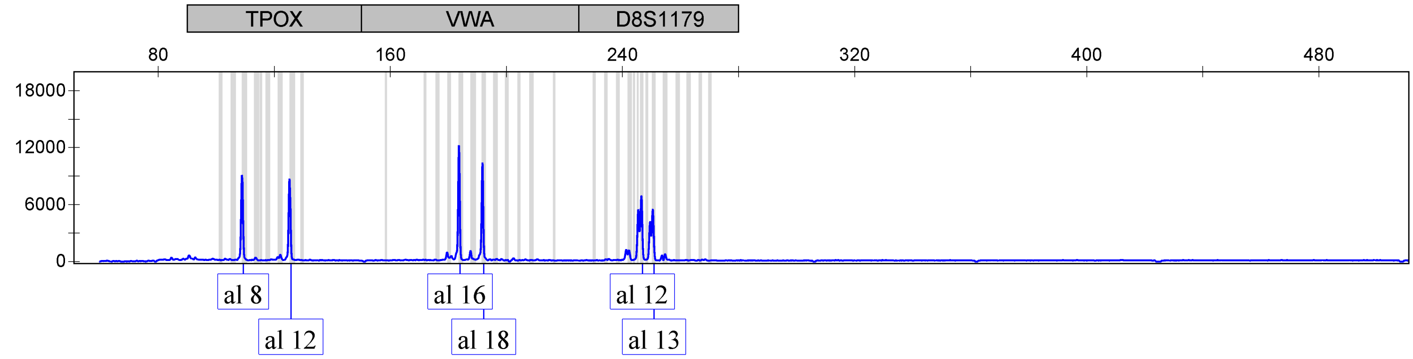

GeneMapper 4.0

xibaojianding-201608

Sample File	Sample Name	Panel	OS	SQ
77_E10_XiBaoJianDing0816-0818.fsa	BGC-823	STR Profile 4-dup		

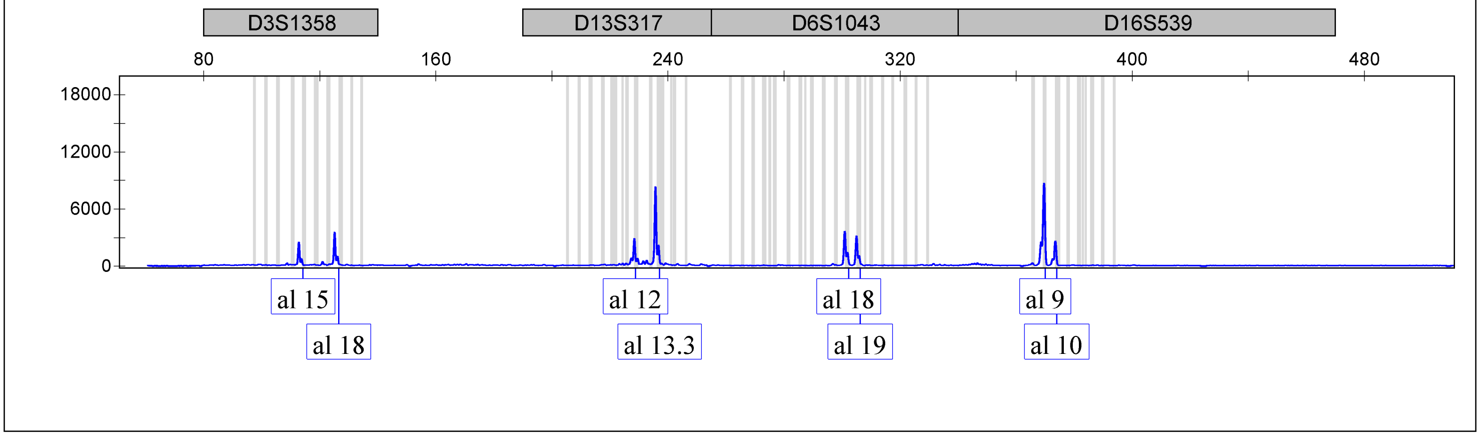

Sample File	Sample Name	Panel	OS	SQ
93_E12_GXGZ0804-12(1-11)XBJD081612-0819.fsa	BGC-823	STR Profile 5-dup		

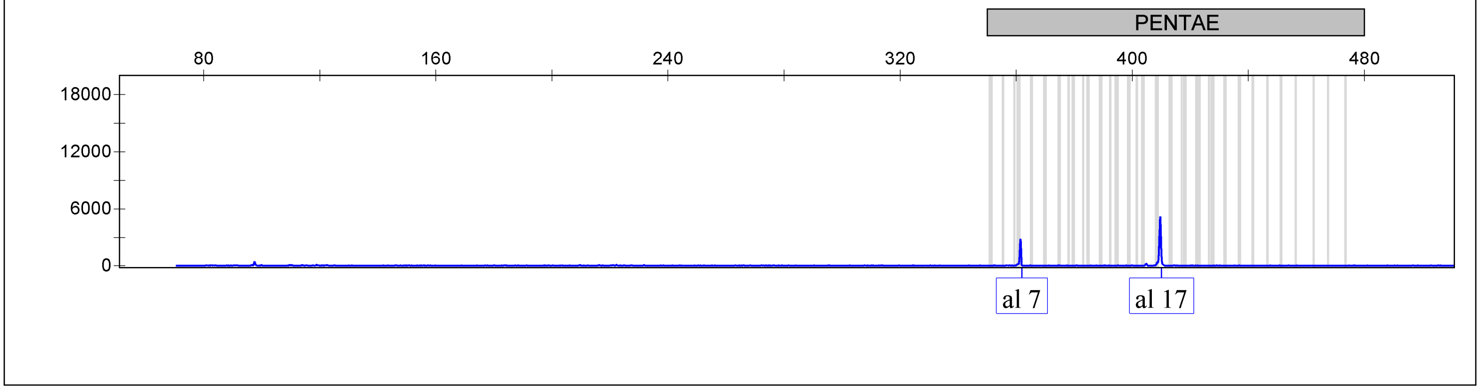

Sample File	Sample Name	Panel	OS	SQ
13_E02_XiBaoJianDing-0822.FSA	BGC-823	STR Profile 6-dup		

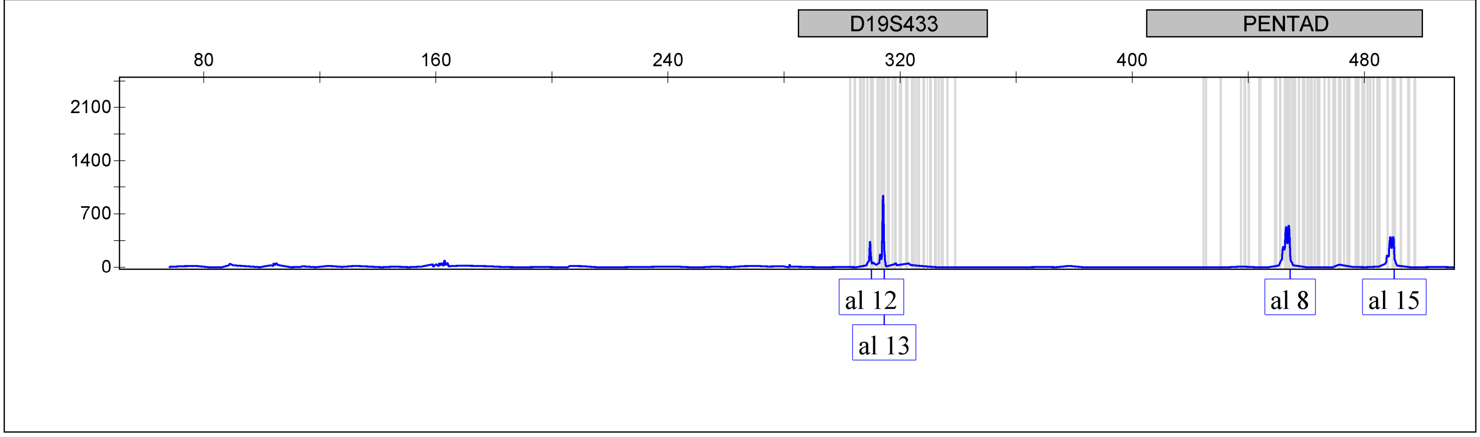

Supplement: Supplementary file 1 [file Supplementaryfile1.pdf]
